# Catalogue of drought events in peninsular Spanish along 1916–2020 period

**DOI:** 10.1038/s41597-024-03484-w

**Published:** 2024-06-27

**Authors:** Víctor Trullenque-Blanco, Santiago Beguería, Sergio M. Vicente-Serrano, Dhais Peña-Angulo, Carlos González-Hidalgo

**Affiliations:** 1 Estación Experimental de Aula Dei, Consejo Superior de Investigaciones Científicas (EEAD-CSIC), 50059 Zaragoza, Spain; 2 Instituto Pirenaico de Ecología, Consejo Superior de Investigaciones Científicas (IPE-CSIC), 50059 Zaragoza, Spain; 3 Departamento de Geografía y Ordenación del Territorio, Universidad de 50009, Zaragoza, Spain; 4 Instituto Universitario de Ciencias Ambientales (IUCA), Universidad de Zaragoza, 50009 Zaragoza, Spain

**Keywords:** Environmental health, Atmospheric dynamics

## Abstract

We leveraged the most extensive and detailed gridded database of monthly precipitation data across the Spanish mainland (MOPREDAScentury), encompassing 1916–2020 time period, to pinpoint the most severe drought events within this timeframe and analyse their spatio-temporal dynamics. To identify these events, we employed the Standardized Precipitation Index (SPI) at a 12-month timescale. Drought events were identified as periods of at least three months where significantly dry conditions affected 20% or more of the study area, defined as grid cells with SPI values lower than −0.84. Our analysis revealed a total of 40 major drought events. Our catalogue contains detailed information on each episode’s spatial extent, duration, severity, and spatio-temporal dynamics. The analysis of the propagation patterns of the events unveils substantial heterogeneity, implying that droughts stem from diverse atmospheric mechanisms, further influenced by complex local topography. The open-licensed drought database serves as a valuable resource. It not only facilitates exploration of drought onset and evolution mechanisms but also aids in assessing drought impact on agricultural and other socio-economic sectors.

## Introduction

Droughts are among the most impactful climatic phenomena, affecting both natural systems and human societies^1,2^. Over the past century^3^, they have led to millions of deaths and substantial economic loss^4^. Additionally, they have caused numerous environmental impacts^5^ and are responsible for the recurrent challenge of supplying water to population centers, agriculture, and various human activities^6,7^.

Droughts have a meteorological origin and manifest over time and space, influencing the entire hydrological cycle and various socio-economic sectors^8^. Their complexity arises from the intricate interactions between climatic conditions^9^, vegetation, soil recharge processes, and the resulting deficits across different components of the hydrological cycle, including surface flows, reservoirs, and groundwater reserves^10^.

Despite numerous factors influencing drought occurrence and severity, the primary factor driving drought remains the deficit of precipitation^11^. The combination of time and magnitude of this deficit can lead to flash droughts^12,13^ or conversely, megadroughts, characterized by their prolonged duration over time and extensive spatial coverage, or even a combination of both^14,15^.

Drought is virtually present everywhere on the planet, but it becomes particularly relevant in dry climates with chronic water deficits. Regions experiencing high inter-annual rainfall variability, coupled with dry summer seasons, like Mediterranean climate areas^16^, are especially susceptible to drought impacts, especially when precipitation deficits are combined with extreme heat. Furthermore, the situation is exacerbated when these conditions coincide with high demands for water resources.

**Fig. 1:**
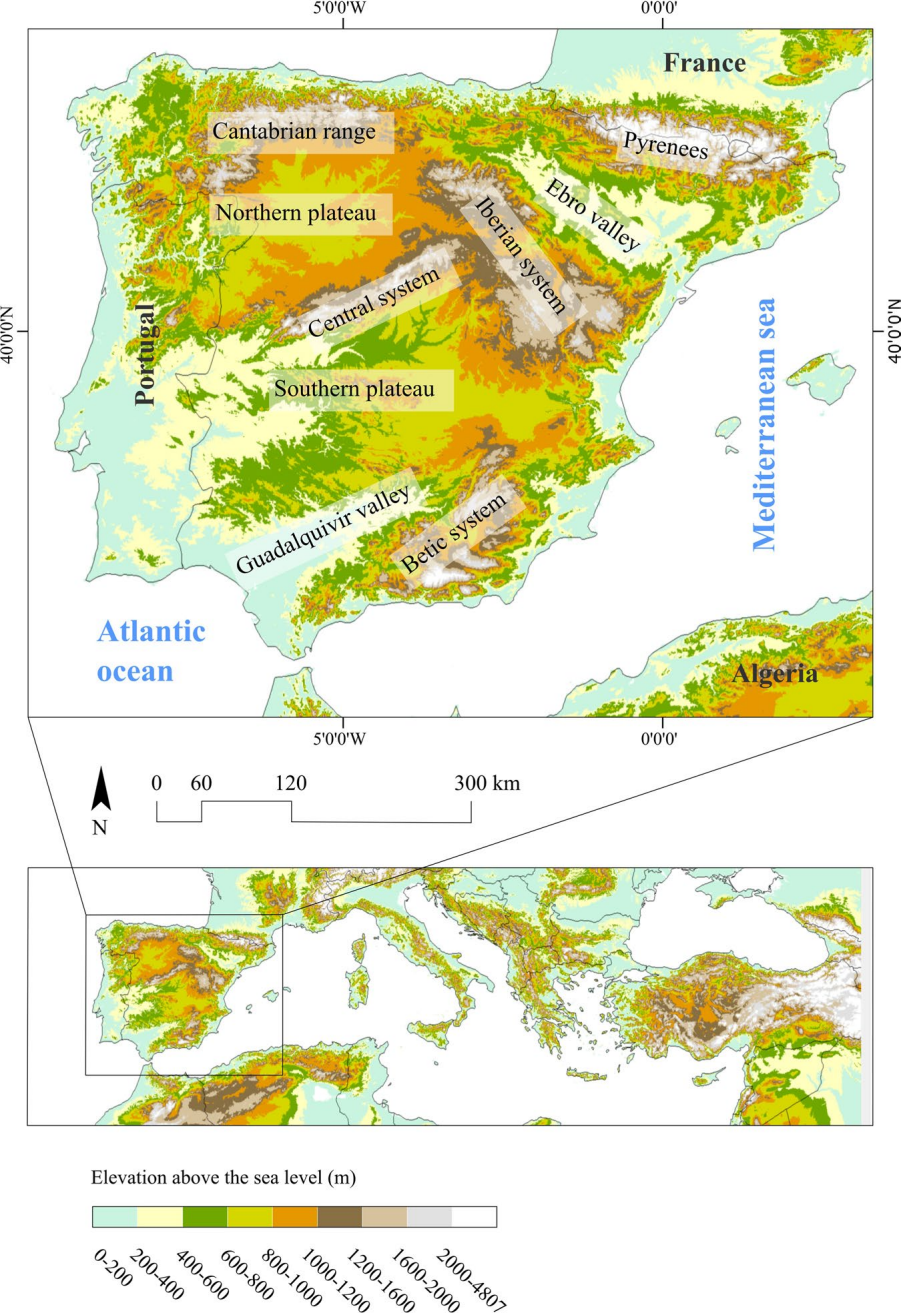
Spanish mainland location in Mediterranean basin, and spatial alignment of the main orographic elements mentioned in the text.

In the western Mediterranean basin, particularly on the Iberian Peninsula (Fig. 1), the historical recurrence of drought events has been well-documented over recent centuries through various sources^17,18^. This pattern persists into the instrumental period since the mid-19th century^19–21^. Recent studies have analyzed the evolving nature of drought events using different indices^22^, examined the varying probabilities of spatial drought occurrence based on their duration and magnitude^23^ and even identified severe historical events using low spatial resolution data^24^. These studies collectively underscore the pronounced spatial and temporal fluctuations characterizing droughts in the region^25^.

Despite the extensive analysis of drought occurrence in Spain from diverse angles, a significant gap remains in understanding the spatio-temporal variations in the characteristics of specific drought events. Also, there’s a need for a comprehensive, long-term digital drought catalog that could serve as a tool for comprehending the spatio-temporal patterns of the phenomenon and its multifaceted impacts (ranging from agricultural and hydrological to environmental and socioeconomic). Addressing this gap is a necessary precursor to the development of drought early warning systems. Hence, this study introduces a comprehensive catalog of drought events in the Spanish mainland, utilizing the new MOPREDAScentury gridded precipitation database covering the period 1916–2020^1^. This novel resource enables the creation of a robust, long-term evaluation of droughts with a high spatial resolution of 10x10 km. The dataset is readily accessible at 10.20350/digitalCSIC/15446^3^, and has been used already to study the precipitation long-term trends^26^ and changes in seasonal precipitation regimes over Spain^27^.

## Results

### Time evolution of the SPI-12 and determination of drought events

**Fig. 2:**
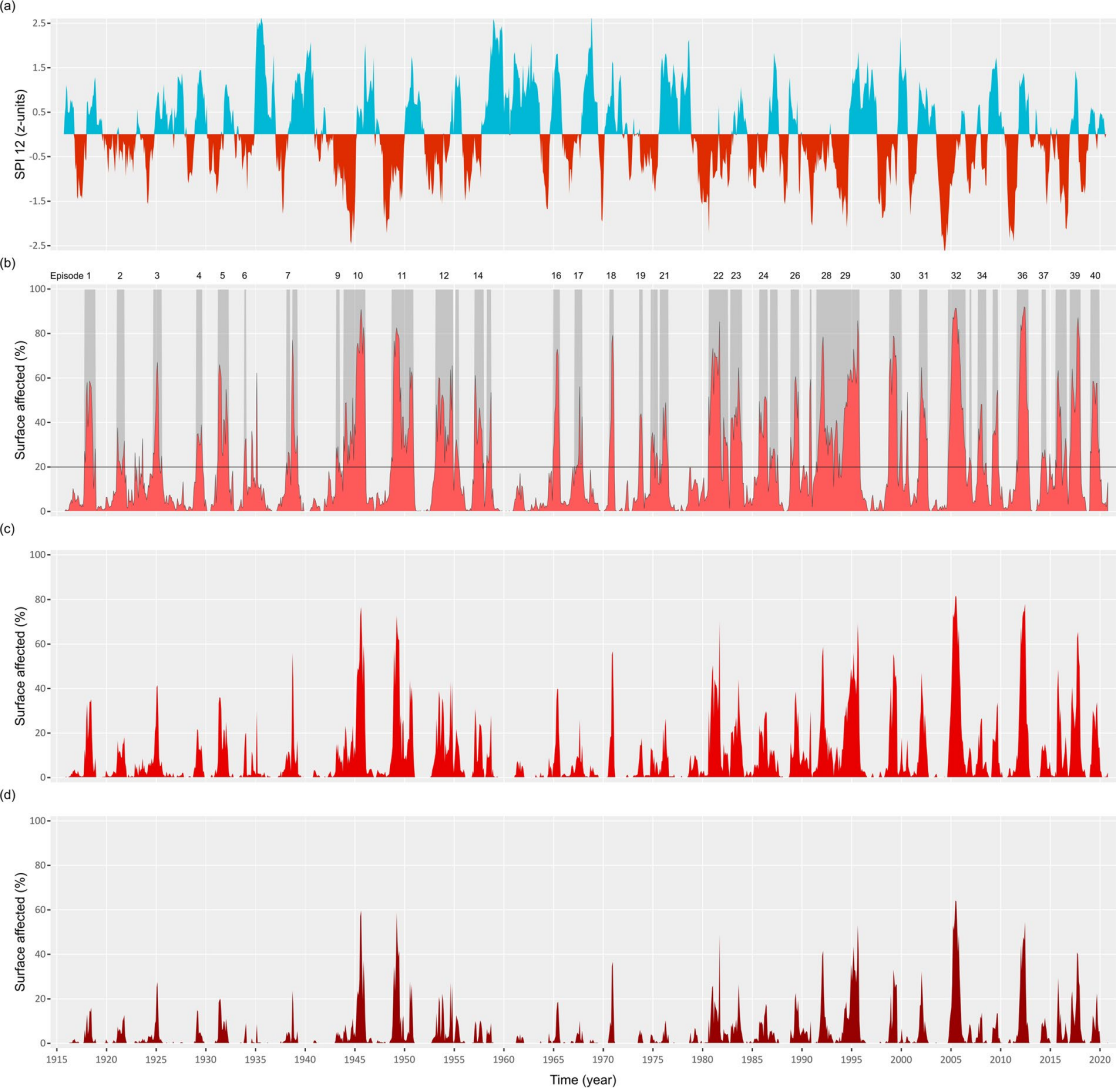
Identification of drought events: time series of the 12-months SPI for the whole study area (**a**); time series of the fraction of the study area under drought conditions according to different thresholds: SPI12 <−0.84 (**b**) SPI12 <−1.28 (**c**) and SPI12 <−1.65 (**d**). The horizontal line at 20% in (**b**) indicates the threshold used for identifying the drought events, which are highlighted by a grey shade and numbered.

The temporal evolution of the 12-month SPI across the entire Spanish mainland exhibits notable temporal variability (Fig. 2a). However, distinct periods characterized by pronounced and enduring drought conditions are evident. Noteworthy instances include the years 1946, 1948/1949, 1994/1995, 2005, and 2012, which stand out as prominent examples of substantial drought periods. In contrast, the decades spanning from 1910 to 1940 and from 1950 to 1980 were relatively unaffected by prolonged and highly severe droughts.

Figure 2b illustrates the temporal evolution of the area impacted by drought conditions, as defined by the specified threshold (SPI-12 <−0.82). By applying the predefined surface threshold (> 20% of affected grid cells), we identified a total of 40 drought events spanning the years from 1916 to 2020. These events are distributed across the entire study duration, encompassing a total of 495 months; that is, 39% of the total period of analysis. For comprehensive information on this dataset and the application of the methodology used for event identification, see Supplementary material.

Furthermore, Fig. 2 also presents the surface affected by more stringent drought thresholds (Fig. 2c,d). The temporal evolution of these two variables closely resembles that shown in Fig. 2b, with the anticipated reduction in the affected surface, and shows that the selection of a specific threshold level didn’t significantly alter the identification of the most prominent drought events.

An interesting observation is that, when considering the time series of inter-arrival times (the time between the start of one drought event and the beginning of the next), the events appear to be over-dispersed. This is evident from the dispersion index, which is calculated as the variance divided by the mean, resulting in a value of 9.95. This value significantly deviates from unity (p-value <0.01), which would characterize a Poisson process. In simpler terms, this suggests that the occurrence of drought events is not random over time. Instead, they tend to happen in clusters or sequences, with some events occurring closely in time to each other, followed by longer periods without drought conditions. This pattern of clustering could have important implications for understanding the temporal dynamics of drought in the studied region.

### Drought events characterisation

**Fig. 3:**
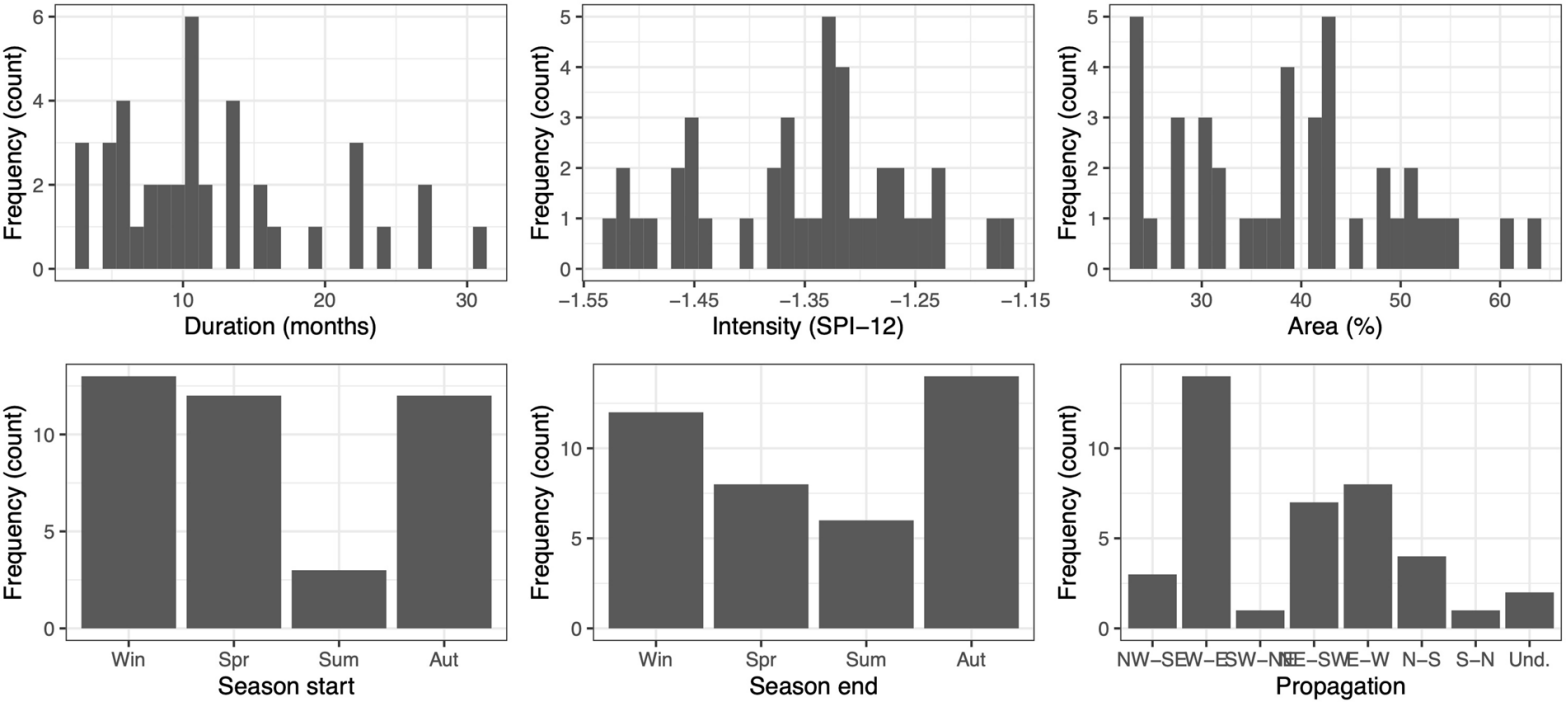
Histograms of the main characteristics of the drought events.

Table 1 offers a comprehensive overview of the key attributes of the 40 identified drought events, encompassing seasonality, duration, average SPI-12 values, total affected area, drought severity, and spatial propagation. For a graphical summary of the main characteristics of the events refer to Fig. 3.

**Table 1:** Catalogue of drought events, with their main characteristics.

Event number	Start-End	Seasonality (start-end)	Duration (months)	Mean intensity (SPI-12)	Area affected (% grid cells)	Months with area >50%			Propagation
						−0.84	−1.28	−1.65	
						mild	moderate	severe	
1	12.1917-01.1919	Win-Win	14	−1.28	39	5	_	_	NW-SE
2	03.1921-12.1921	Spr-Win	10	−1.44	24	_	_	_	N-S
3	11.1924-09.1925	Aut-Aut	11	−1.32	37	3	_	_	NE-SW
4	03.1929-10.1929	Spr-Aut	8	−1.37	32	_	_	_	NE-SW
5	05.1931-06.1932	Spr-Sum	14	−1.33	43	5	_	_	E-W
6	01.1934-03.1934	Win-Spr	3	−1.36	30	_	_	_	W-E
7	04.1938-08.1938	Spr-Sum	5	−1.33	23	_	_	_	Undefined
8	10.1938-05.1939	Aut-Spr	8	−1.29	43	3	1	_	W-E
9	04.1943-08.1943	Spr-Sum	5	−1.32	24	_	_	_	E-W
10	01.1944-03.1946	Win-Spr	27	−1.38	52	12	6	2	W-E
11	11.1948-01.1951	Aut-Win	27	−1.45	51	13	6	1	NE-SW
12	04.1953-01.1955	Spr-Win	22	−1.34	43	7	_	_	E-W
13	03.1955-08.1955	Spr-Sum	6	−1.53	27	_	_	_	E-W
14	03.1957-02.1958	Spr-Win	12	−1.25	39	2	_	_	W-E
15	06.1958-11.1958	Sum-Aut	6	−1.3	32	1	_	_	NE-SW
16	02.1965-10.1965	Win-Aut	9	−1.28	48	3	_	_	W-E
17	04.1967-01.1968	Spr-Win	10	−1.23	27	1	_	_	NE-SW
18	10.1970-03.1971	Aut-Spr	6	−1.47	54	3	2	_	E-W
19	10.1973-02.1974	Aut-Win	5	−1.24	36	_	_	_	NE-SW
20	12.1974-08.1975	Win-Sum	9	−1.17	27	_	_	_	SW-NE
21	11.1975-09.1976	Aut-Aut	11	−1.23	30	_	_	_	W-E
22	10.1980-09.1982	Aut-Aut	24	−1.33	49	12	2	_	W-E
23	12.1982-02.1984	Win-Win	15	−1.35	41	2	_	_	W-E
24	11.1985-09.1986	Aut-Aut	11	−1.38	41	3	_	_	E-W
25	12.1986-09.1987	Win-Aut	19	−1.26	23	_	_	_	N-S
26	01.1989-11.1989	Win-Aut	11	−1.4	39	4	_	_	NW-SE
27	12.1990-02.1991	Win-Win	3	−1.18	46	2	_	_	W-E
28	08.1991-05.1993	Sum-Spr	22	−1.33	38	4	2	_	W-E
29	06.1993-12.1995	Sum-Win	31	−1.46	48	14	3	_	S-N
30	12.1998-03.2000	Win-Spr	16	−1.32	51	10	2	_	E-W
31	12.2001-10.2002	Win-Aut	11	−1.45	43	3	_	_	NE-SW
32	11.2004-08.2006	Aut-Sum	22	−1.52	61	13	9	6	W-E
33	01.2007-03.2007	Win-Spr	3	−1.49	23	_	_	_	Undefined
34	11.2007-09.2008	Aut-Aut	11	−1.27	30	_	_	_	N-S
35	05.2009-11.2009	Spr-Aut	7	−1.36	41	2	_	_	W-E
36	10.2011-12.2012	Aut-Win	15	−1.5	63	10	7	1	N-S
37	04.2014-09.2014	Spr-Aut	6	−1.51	25	_	_	_	E-W
38	09.2015-10.2016	Aut-Aut	14	−1.33	34	3	_	_	W-E
39	02.2017-03.2018	Win-Spr	14	−1.45	55	8	4	_	NW-SE
40	03.2019-02.2020	Spr-Win	12	−1.32	43	5	_	_	W-E

Mean intensity and area affected refer to the grid cells with values of the SPI-12 lower than −0.84.

Of particular interest is the seasonality aspect. It’s worth highlighting that a significant majority of the drought events initiated in winter (33%), spring (30%), or autumn (30%). In contrast, only a small fraction of events commenced in the summer (7%). Similarly, a large proportion (85%) of the events also ended in these same seasons. A large variability is observed between the beginning and the end of the events. Notably, what stands out is the absence of any events that commenced during the spring or summer seasons and concluded within the same seasons. In contrast, events that originated in winter could conclude in any of the four seasons, showcasing a broader variability in their endpoints. On the other hand, drought events that began in autumn exhibited a certain tendency to conclude also in autumn.

Concerning the duration of the events, there are seven instances of prolonged droughts that extended beyond 20 months. Additionally, 17 events persisted for a duration of 12 months or more. Interestingly, the duration of the drought episodes doesn’t seem to exhibit a direct correlation with the timing of their occurrence. Thus, the longest episodes are ranked as number 10 (lasting 27 months), number 11 (also 27 months), and number 29 (stretching over 31 months). These events unfolded during 1944–1946, 1948–1951, and 1993–1995, respectively. Further noteworthy instances marked by extended duration include events numbered 12, 22, 28, and 32, each spanning 22 months.

The average area affected by drought ranged between 23% of the grid cells (event 7) to 63% (event 36). The average area affected did not seem to show a direct correlation with the timing of occurrence, the event duration, nor average intensity. Notably, in seven instances, the area affected by drought conditions surpassed 50% of the grid cells for at least ten months. Taking the largest event, number 29, as an example, drought impacted 50% of the grid cells for 14 consecutive months. Another noteworthy episode with an extended duration is event number 32, during which drought conditions extended to over 50% of the grid cells for 13 months.

In terms of their global severity of the drought events, it resulted from the interplay of their duration, average SPI, and affected area. Events 32 and 29 particularly stand out, marked by an average SPI-12 of −1.52 and −1.46, respectively, and affecting 61% and 48% of the grid cells. In both instances, a convergence of factors such as substantial accumulated rainfall deficit, prolonged duration (22 and 31 months), and extensive spatial coverage contributed to their remarkable severity. Similarly, event number 36, while shorter in duration (15 months), displayed significant intensity (average SPI-12 of −1.50) and wide coverage (63% of grid cells). This event witnessed eight months surpassing the drought threshold in 50% of the grid cells. Events 10 and 11 also belong to this group. While their SPI-12 values were somewhat less intense, their severity stemmed from their extended duration (27 months) and spatial extent (over 50% of grid cells). Additionally, it’s important to highlight that a few events registered average SPI-12 values that surpassed even the most severe events mentioned earlier. However, their duration or spatial extent was less pronounced. This outcome underscores the considerable variability in the manifestation scales of drought events.

The analysis of the spatial patterns of the drought events merits a dedicated section. The main propagation direction of the events sere determined visually, upon inspection of the sequence of monthly maps of the SPI-12 during each event. This analysis enabled establishing general patterns of drought propagation. These patterns present notable variability, highlighting the complexity of droughts over the study area. The main conclusion of this analysis is that a vast majority of events exhibited a zonal nature (west-to-east, or east-to-west). Considering their origin (region affected during the first or earlier months of the event), they can be categorized into either having an Atlantic origin (NW, W, and SW) accounting for 45% of the events, or a Mediterranean origin (NE, E, and SE) comprising 38%. Instances of meridional origins (N and S) represent a minority, at 17%. It’s important to note that within the Atlantic-origin events, a significant portion have a distinct westward origin (35%). Meanwhile, Mediterranean-origin events predominantly stem from the east (20%) and the north-east (18%).

Only a limited number of events remained confined within their initial regions (labelled as ‘Undefined’ in Fig. 3). The typical pattern involved expansion into adjacent areas, thus establishing the principal trajectory or direction of this progression. Interestingly, the propagation trajectory of the events reverses the pattern described above for the origin of the events. Thus, about 45% of the events exhibit a Mediterranean direction (towards the east), whereas 38% have an Atlantic direction (towards the west). Notably, a considerable portion of the propagations involve an inverse origin-destination relationship between the Atlantic and Mediterranean regions.

### Drought events analysis

For the remainder of this section, we provide an in-depth analysis of a curated set of drought events. The purpose of this event selection is to showcase the wide spectrum of droughts that define the study region. The presentation of the events adheres to a consistent structure, elaborated comprehensively in the analysis of the first event.

#### Event 10: 1944-01/1946-03

**Fig. 4:**
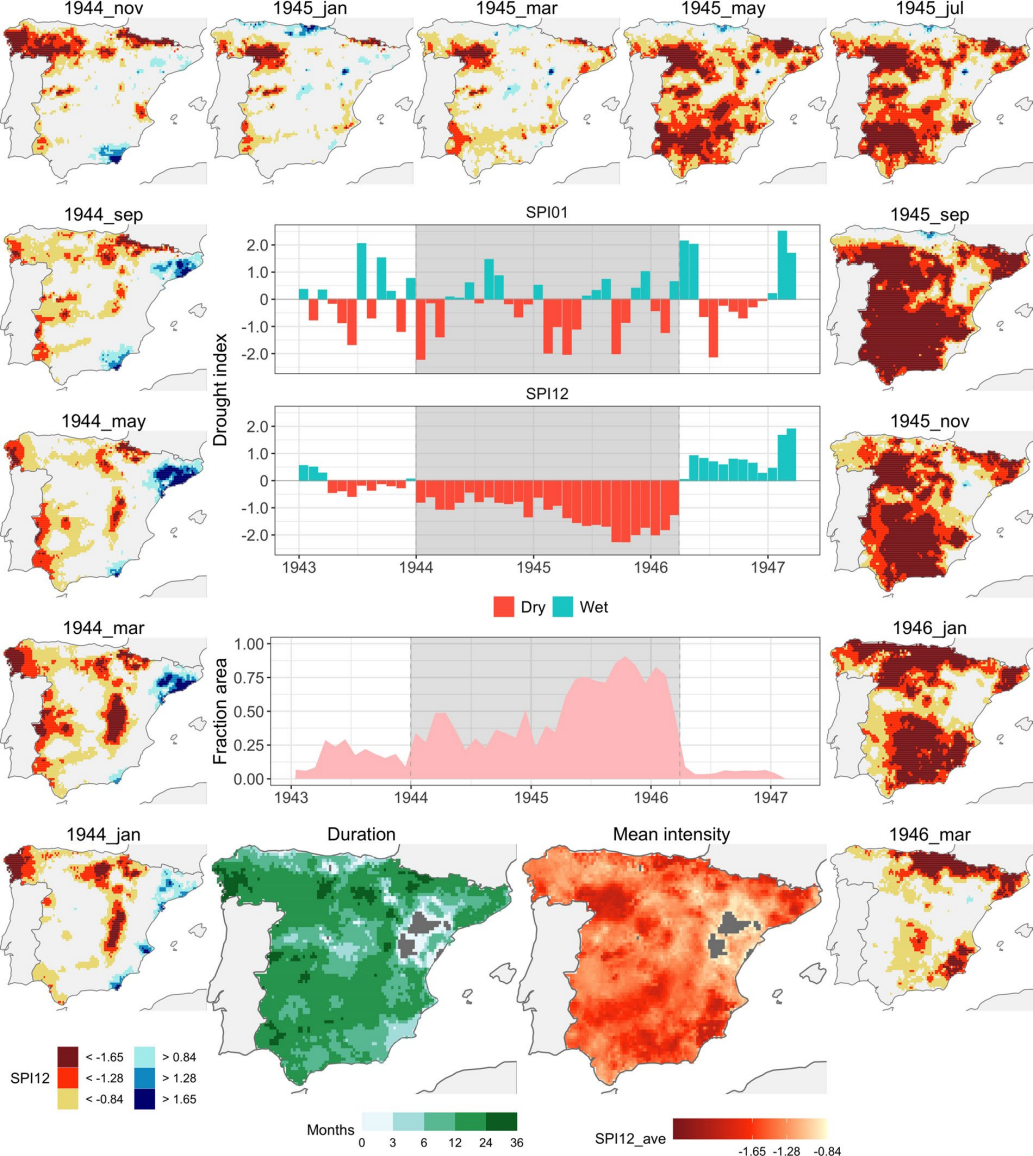
Event 10 (1944-01/1946-03). Main plots (central column): time series of the global SPI01, SPI12 and area under drought, with indication of the drought episode (shaded area); integral maps of drought duration and mean drought intensity (SPI12) during the episode. Miniature maps (surrounding the central column): sequence of monthly SPI-12 during the event, illustrating the spatial propagation of the area under drought at selected moments during the drought episode.

The first event, identified as number 10 in the drought catalogue, extended from January 1944 to March 1946, encompassing a total duration of 27 months. Figure 4 presents a composition of plots featuring key components for event analysis. The peripheral maps that surround the Fig. portray a chronological sequence of monthly SPI-12 values at different times throughout the event. The maps are organized in a clockwise fashion. The colour legend employed delineate areas experiencing drought conditions as per the thresholds outlined in the Methods section, while contrasting with areas displaying wetter-than-normal conditions. The central portion of the Fig. houses three time series plots. These encompass the global SPI-1, the SPI-12, in both cases computed from the time series of average precipitation over the whole area, and the proportion of grid cells affected by drought. The shaded region within these plots identifies the event’s duration, in accordance with the criteria described in the Methodology section. Lastly, two maps positioned at the bottom of the central column of the Fig. depict the spatial distribution of drought duration and intensity (average SPI-12) for grid cells subjected to drought conditions (SPI-12 <−0.84).

The sequence of monthly SPI-1 values showcases an irregular pattern characterized by alternating wet and dry months. However, due to the high variability of this index, it doesn’t distinctly delineate the period as a drought. Contrastingly, the accumulation over a 12-month period, represented by the SPI-12, provides a much clearer depiction of the dry period. Examining the time series of the area affected by drought, one can observe the gradual expansion of the drought. This expansion adheres to the criteria of 20% of grid cells affected by January 1944, eventually culminating in over 85% of the area affected during the latter half of 1945. Subsequently, this impact significantly receded in February and March of 1946.

Over the course of its progression, this event affected the entire study area, excluding only a small region within the centre of the Ebro Valley and the Iberian Range. This instance exemplifies an eastward-westward propagation. Both in terms of its duration and the extent of the area affected, this event stands as one of the most severe encountered by the contiguous Spanish land during the analysed period. In fact, this was the last drought that caused widespread famine and mortality in the country^28,29^. Thus, in more than 50% of grid cells the conditions of moderate drought persisted for a minimum of 12 months. Regions exhibiting very low mean SPI-12 values are observed across extensive inland areas. Areas displaying less severe drought impact were situated along the northern coast and the previously mentioned region encompassing the central and lower Ebro Valley.

#### Event 13: 1955-03/1955-08

**Fig. 5:**
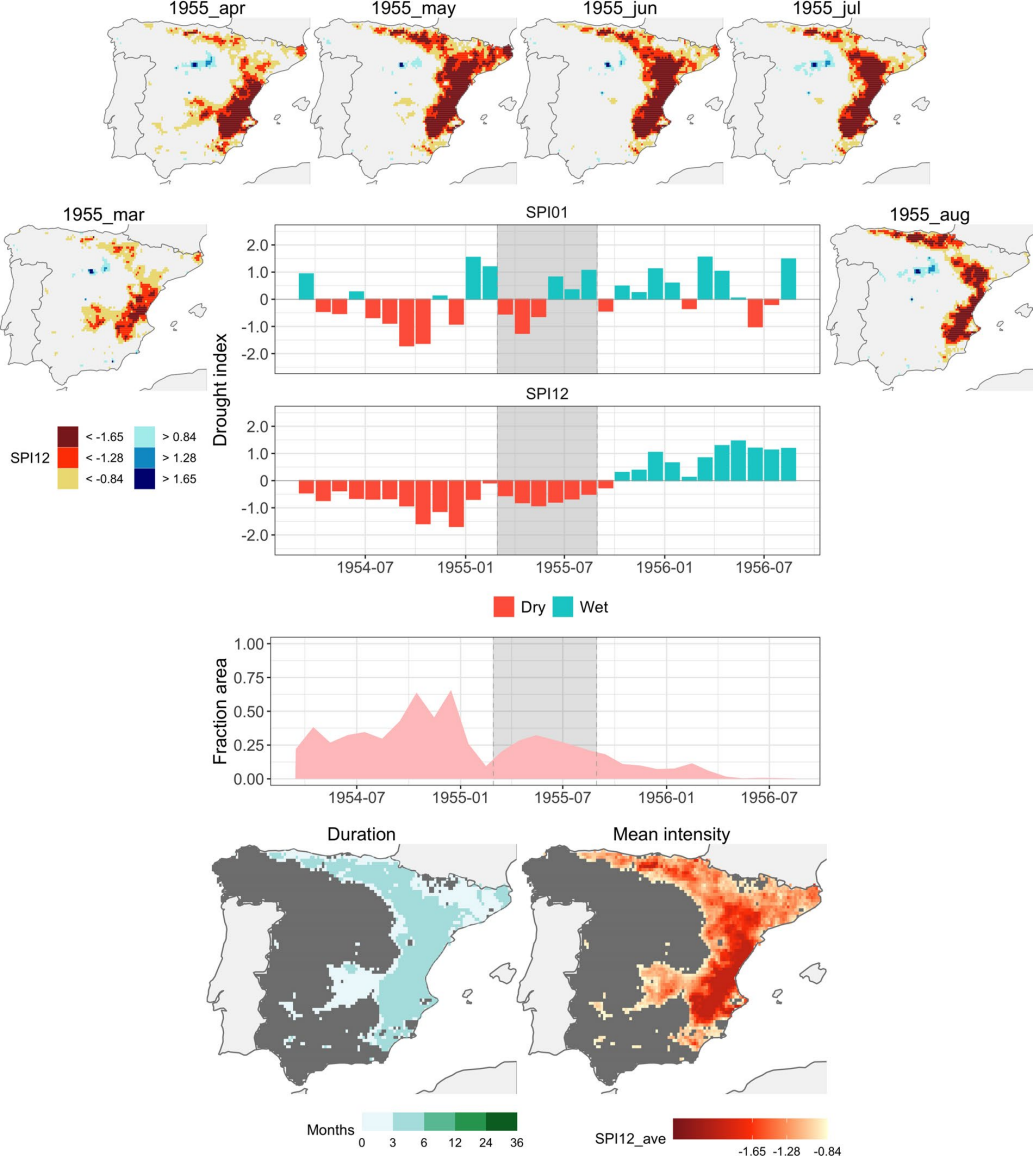
Event 13 (1955-03/1955-08). See Fig. 4 for explanation.

The next event, designated as number 13, is featured in Fig. 5. In contrast to the preceding case, it was relatively brief, surpassing the 20% grid threshold for only six months. However, its distinction lies in the intensity of SPI-12 values within the affected regions, which ranks among the highest throughout (with an average SPI of −1.53). An important fact to acknowledge is that this event commenced merely two months after the conclusion of the preceding event, number 12. This time span is separated by just a single month (February 1955), during which 9.4% of the grid cells were affected by drought. This situation nearly meets the criteria for the two events to be considered as a single occurrence. Nonetheless, given the distinct spatial characteristics of these two events, we considered that maintaining their separation is appropriate.

The impact of this event was primarily concentrated in the Mediterranean coastal region, where the strongest drought intensities were recorded, permeating through the Ebro Valley towards the northern Atlantic coast.

#### Episode 36: 2011-10/2012-12

**Fig. 6:**
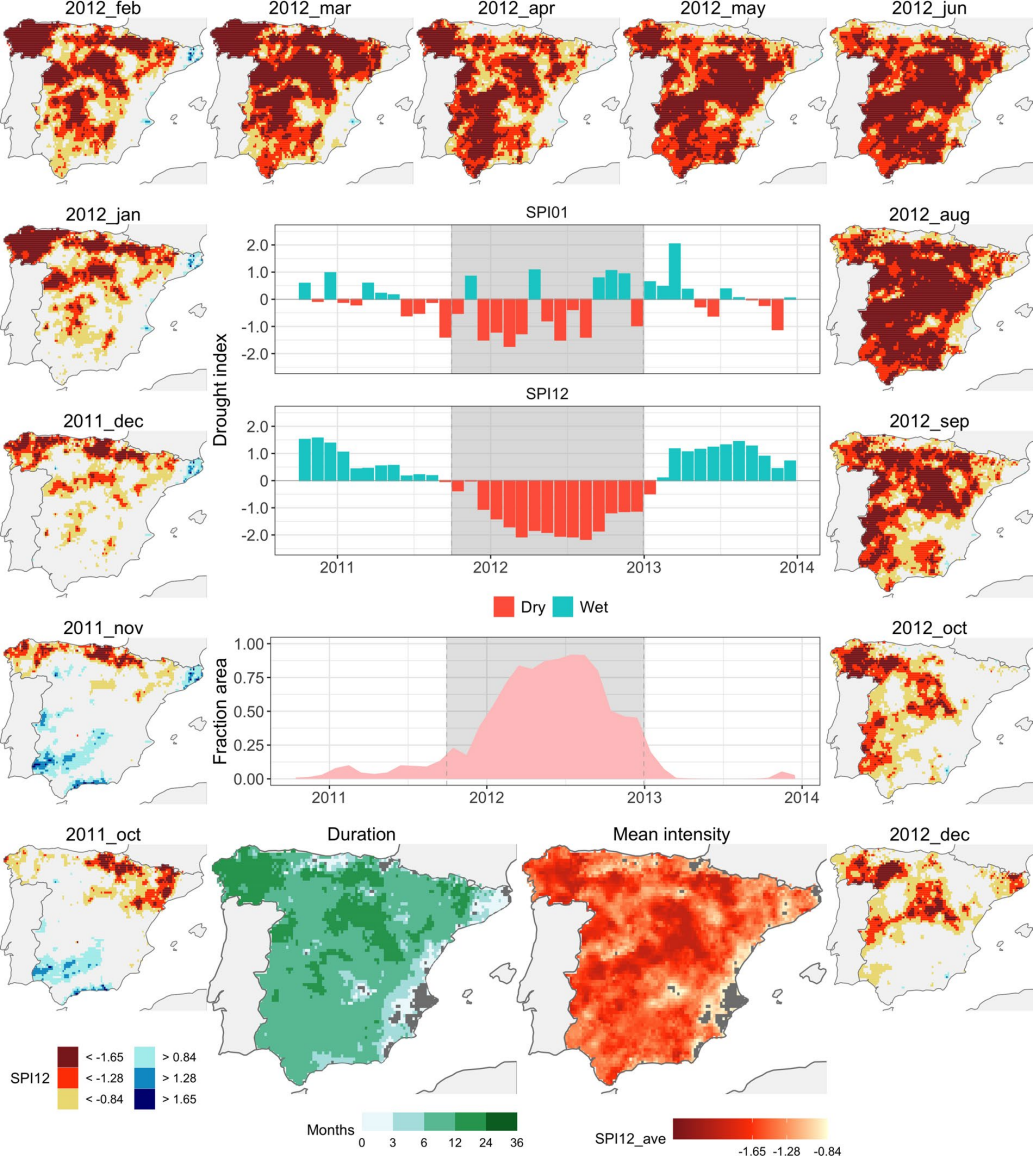
Event 36 (2011-10/2012-12). See Fig. 4 for explanation.

Event 36 ranks as one of the lengthiest (spanning 15 months) and most spatially extended within the collection (Fig. 6). The requisites for attaining the status of a drought event (involving 20% of the grid cells) were met in October of 2011. The affected region underwent a progressive increase, sustaining over seven months with more than 50% of the grid cells impacted in 2012. Subsequently, drought conditions underwent gradual mitigation during the latter half of 2012, ultimately concluding in December of that year.

**Fig. 7:**
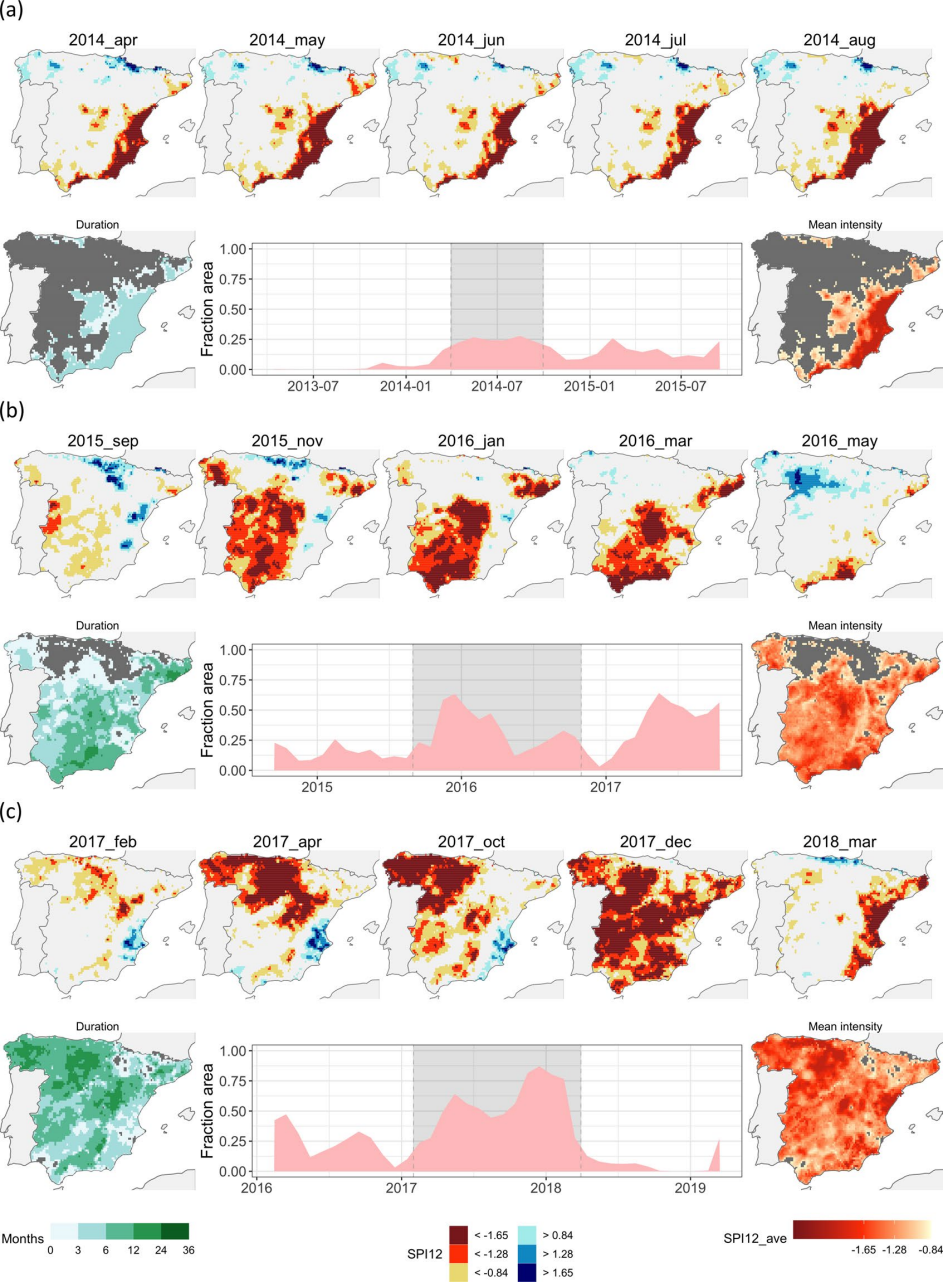
Three recent droughts events: number 37 (2014-04/2014-09), (**a**) number 38 (2015-09/2016-10), (**b**) and number 39 (2017-02/2018-03), (**c**) Miniature maps of the SPI12 at selected moments (upper row; same as in Fig. 4); time series of the area under drought and integral maps of drought duration and mean drought intensity during the episode (bottom row).

This event encompassed almost the entirety of the study area, with the strongest severity in the centre (Central and Iberian Systems) and north-west (Galicia) regions. Comparatively, milder impact was experienced along the Mediterranean coast and the north-eastern region (Catalonian inner basins).

The initiation of this episode took place in the north-east of the study area. Here, strong but localized deficits were observed along the Ebro basin. Subsequently, the drought conditions spread along the northern coast, progressively advancing to the west and southwards, leaving only a limited unaffected stretch along the Mediterranean coast.

#### Three recent drought events

Figure 7 offers a condensed overview of three recent events, labelled as 37, 38, and 39, each of which is depicted with a concise propagation sequence.

Event 37 spanned a mere six months, from April to September 2014. It exhibited significant severity (with an average SPI-12 of −1.51) and predominantly impacted the south-eastern portion of the study area along the Mediterranean coast. There was limited extension towards the upper Tagus Valley. Interestingly, this event displayed almost negligible spatial propagation.

Event 38, which began approximately a year later in September 2015 and concluded in October 2016, persisted for a total of 14 months. It predominantly affected the central and southern regions of the study area, with a comparatively milder intensity (mean SPI of −1.33). In contrast, most of the northern region remained unaffected. This event showed a west-to-east (W-E) propagation pattern, concluding in the eastern half of the study area.

Lastly, event 39 spanned 14 months, stretching from February 2017 to March 2018. It impacted virtually the entire study region, particularly the central and north-western areas. This event exhibited a distinct north-west to south-east (NW-SE) propagation, peaking at the close of 2017, with over 75% of the grid cells affected. Interestingly, during the first half of the event, the south-eastern region consistently experienced wet conditions, which transitioned into very dry conditions during the event’s second half and its concluding stages.

## Discussion

Past investigations have noted that drought indices spatial and temporal dynamics across mainland Spain are typified by high variability. This leads to a marked non-synchronization of drought events, with only a sparse few managing to impact the entire region^19,23^. While several studies have pinpointed several extreme drought events in Spain^24,30^, they did not encompass the combination of high spatial resolution, extended temporal scope, and comprehensive spatio-temporal attributes of drought events within the region, as we have shown here. Thanks to the new dataset presented herein, the identification of major drought events that have affected mainland Spain across the past century has become feasible. This dataset has also allowed for the comprehensive delineation of these events’ key characteristics, particularly their spatio-temporal propagation patterns. The events catalogued exhibit a rich array of characteristics concerning their spatial propagation patterns, reinforcing some of the trends previously documented for the period 1960–2012^30^.

The occurrence of drought events appears to align with the primary routes of moisture advection into the Iberian Peninsula^31^, while also being influenced by the topography, which distinctly shapes rainfall distribution^27,32^. Broadly, the absence of precipitation stemming from an Atlantic origin leads to events commencing in the west, gradually advancing towards the east. Conversely, the absence of Mediterranean precipitation leads to events initiating on the eastern coast and then progressing inland with an east-west propagation. These local situations could be easily linked to broader-scale atmospheric circulation mechanisms influencing the region. These include the North Atlantic Oscillation, the Mediterranean Oscillation, and the Western Mediterranean Oscillation, among others, all of which have been extensively researched in terms of their regional effects on precipitation^33–36^. Similarly, they could also be linked to the distinct roles of various weather types^37–39^. This overarching mechanisms of atmospheric circulation on hemispheric or global scales^40,41^ collectively yield a wide spectrum of drought events. These range from highly localized occurrences to rarer ones capable of affecting the entire Iberian Peninsula^42^.

Given this intricate scenario, the current long-term database provides an unprecedented opportunity to assess the physical mechanisms underpinning drought events in mainland Spain. Prior studies focused on the physical mechanisms shaping specific drought events have underscored the diverse atmospheric triggers for droughts. They’ve also highlighted how different events can be characterized by highly distinct mechanisms, as evidenced in the cases of the droughts in 2005^42^, 2012^43^, and 2016^44^. The marked diversity in the spatial origin, propagation, and seasonality of droughts uncovered in our study supports the idea that a range of distinct physical mechanisms could govern the occurrence of drought events. This aspect warrants further investigation, as an enhanced understanding of the complex processes behind droughts in Spain could bolster the capability for drought prediction in the region. This, in turn, could mitigate the substantial uncertainties associated with dynamic modelling^45–47^.

The recent generation of drought impact databases from different perspectives^48,49^ may allow to relate drought characteristics with a variety of drought impacts and to improve drought adaptation. Also socioeconomic disciplines can be benefited of this data base as several studies have linked drought with economic impacts^48,50,51^, but they are difficult to determine. Recently, Spain has developed drought monitoring systems based on indices^52,53^. The link between thresholds of drought indices and drought impacts would improve the early warning. Nevertheless, for this purpose it is necessary to consider the complex spatio-temporal behaviour that characterises droughts in Spain and this drought event database covers the existing needs about this issue.

In the development of this dataset, the establishment of specific criteria for drought severity, spatial extent, and duration was essential to delineate the beginning and end of drought events. The selection of these threshold values does involve a degree of subjectivity, as alternative thresholds could potentially yield different sets of drought events. In this study, we’ve demonstrated that opting for more stringent drought severity thresholds, corresponding to longer return periods (SPI values of −1.28 and −1.65), doesn’t significantly alter the time series of drought events. Regarding the minimum duration criterion of three months, this choice was made to strike a balance. A lower threshold (one or two months) might have led to the identification of very short-lived events of limited significance, while a higher threshold would have excluded events that were considered relevant. The spatial extension threshold, set at 20% of the grid cells, is the most sensitive among the criteria and could yield notably different outcomes with different values. The choice of this threshold resulted in the identification of 40 events, which accounted for 39% of the months within the study period. This choice was seen as a practical compromise between having too few, short events (with a higher threshold) and very large events encompassing a substantial proportion of the months under study (with a lower threshold).

## Methods

The study is based on the MOPREDAScentury high-resolution gridded precipitation data set^1,2^. This grid encompasses 5219 10 x 10 km^2^ monthly precipitation cells and spans the timeframe from 1916 to 2020. The database integrates original data from the National Climate Data Bank of the Spanish National Meteorological and Climatological Agency (AEMET), along with digitized data from the Annual Summary Books spanning 1916–1950. A comprehensive outline of the process, which includes source amalgamation, quality control, and data processing, is detailed in Beguería *et al*.^1^.

As a first step to identify droughts, we transformed the original data to local precipitation anomalies by using the Standardized Precipitation Index, SPI^54^, at a 12-month time scale (SPI-12). Time series of the SPI-12 index were computed for each grid cell, adhering to the guidelines set by the World Meteorological Organization^55^. Then, identification of drought events involved the integration of three criteria:

1. Severity: for each month during the study period, all grid cells with SPI-12 values lower than −0.84 were labelled as undergoing dry conditions. This threshold corresponds to a 5-year return period, which is conventionally considered as a mild drought^56^. Alternatively, two versions of this dataset were also created using more stringent thresholds: SPI-12 <−1.28 (10-year return period, moderate drought), and SPI-12 <−1.65 (20-year return period, severe drought).
2. Spatial extent: all months entailing more than 20% of the grid cells under drought conditions (SPI <−0.84) were labelled as dry months.
3. Temporal duration: drought events were subsequently identified as sequences of consecutive dry months extending for a minimum duration of three months. Gaps up to three months were allowed within an event if they contained at least 10% of dry grid cells. In a few cases, though, the event was split at the gapping months if there was no spatial coherence between the two sections. Such was the case of events 7-8, 20-21, and 28-29.

For each drought event the following characteristics were determined:

- Duration: event’s total length, in months.
- Seasonality: starting and ending seasons.
- Magnitude: integral (sum) of all the grid cells SPI-12 values lower than −0.84.
- Area affected: spatial extent of the drought averaged over the event, in percentage.
- Spatial propagation: the spatial propagation was determined through the observed trajectory of the area affected by drought during the episode. Thus, the approximate location within the study area of the area affected by drought (N, N-E, E, etcetera) were determined at the beginning and at the end of the event, or if the episode’s development lacked a clear spatial propagation, it was classified as indeterminate.

Additionally, and for illustration purposes, we also computed a time series of the SPI-1 and SPI-12 indices from the monthly precipitation time series resulting from averaging precipitation values over all the grid cells.

### Supplementary information

Supplementary Material

Supplementary table

## Data Availability

All data generated and evaluated in this publication are available at DIGITAL.CSIC repository (10.20350/digitalCSIC/15446^3^). The database consists of one netCDF file and two zipped folders. The first folder includes a descriptive analysis of 40 drought episodes identified according to the criteria detailed on this article. For each episode, it includes the time series of spatially averaged SPI01 and SPI12; the fraction area under drought; the integral maps of the episode according to its duration (number of months) and intensity (average SPI of the cells under drought); and the sequence of maps representing the spatial propagation of drought conditions during the episode. The second folder includes the code necessary to generate the descriptive analysis material. The netCDF file consists of six variables. The first five are the precipitation anomalies (Standardized Precipitation Index at 1, 3, 6, 12 and 36-month time scales (namely ‘SPI-01’, ‘SPI-03’, ‘SPI-06’, ‘SPI-12’ and ‘SPI-36’). The sixth variable is the id code of the drought events (namely ‘evnt’), numbered from 1 to 40. The time dimensions of the netCDF dataset are 1915/01-2020/01.

## Code availability

Code used in the 40 drought events characterization is available at DIGITAL.CSIC (10.20350/digitalCSIC/15446). Code was run using the 2023.06.0 RStudio version.
