# Supplementary material for: Catalogue of drought events in peninsular Spanish along 1916–2020 period
Source: Sci Data. 2024 Jun 27;11:703. doi: 10.1038/s41597-024-03484-w (PMC11211438; doi:10.1038/s41597-024-03484-w)
Supplement: Supplementary file 2 — Supplementary table [file 41597_2024_3484_MOESM2_ESM.docx]

**Supplementary Material.** Determination of drought events according to the surface affected by drought. Red dots represent the months in which 25% of the grid cells are under the threshold of SPI =< -0.84, and yellow dots represent the months in which 10% of the grid cells are under the threshold of SPI =< -0.84. Groups larger than three consecutive months under ‘red’ or ‘yellow’ conditions are considered an event (outlined in the table).
